# Supplementary material for: Mbnl1 and Mbnl2 regulate brain structural integrity in mice
Source: Commun Biol. 2021 Nov 30;4:1342. doi: 10.1038/s42003-021-02845-0 (PMC8633067; doi:10.1038/s42003-021-02845-0)
Supplement: Supplementary file 2 — Supplementary Material [file 42003_2021_2845_MOESM2_ESM.pdf]

Supplementary Material

Mbnl1 and Mbnl2 regulate brain structural integrity in mice

Naomi Sta Maria[1#], Chenyu Zhou[2#], Se Jung Lee[2#], Parvin Valiulahi[2], Xiandu Li[3], Jongkyu Choi[2], Xiaodan Liu[1], Russell Jacobs[1], Lucio Comai[3*], Sita Reddy[2*]

1. Department of Physiology and Neuroscience, Keck School of Medicine, University of Southern California, Los Angeles, California, USA

2. Department of Biochemistry and Molecular Medicine, Keck School of Medicine, University of Southern California, Los Angeles, California, USA

3. Department of Molecular Microbiology and Immunology, Keck School of Medicine, University of Southern California, Los Angeles, California, USA

# These authors contributed equally

* Corresponding authors

Supplementary Method

Development of *Mbnl2*$^{ΔE2/ΔE2}$ mice: To test the role of *Mbnl2* loss in the development of DM1 CNS dysfunction we developed *Mbnl2*$^{loxE2lox/loxE2lox}$ (indicated as *Mbnl2*$^{fl/fl}$) mice in which exon 2 of *Mbnl2* was flanked by lox sites (red arrowheads) [Supplementary Figure 1, a(i-ii)]. Southern blot analysis of targeted 129sv *Mbnl2*$^{+/fl}$ ES cells is shown in Supplementary Figure 1, b. Chimeric animals derived from targeted 129sv ES cells were bred to 129sv wild type animals to derive 129sv *Mbnl2*$^{+/fl}$ mice. 129sv *Mbnl2*$^{+/fl}$ mice were crossed with 129sv transgenic mice expressing the Cre recombinase under the control of the protamine 1 promoter [1] to achieve Lox mediated constitutive deletion of *Mbnl2* exon 2 (*Mbnl2*$^{+/ΔE2}$) (Supplementary Figure 1, aii, aiii). Deletion of *Mbnl2* exon 2 was established by RT-PCR analyses in *Mbnl2*$^{ΔE2/ΔE2}$ mice (Supplementary Figure 1, c). Splice alterations in the forebrain of *Mbnl2*$^{ΔE/ΔE2}$ mice is shown in Supplementary Figure 1, d.

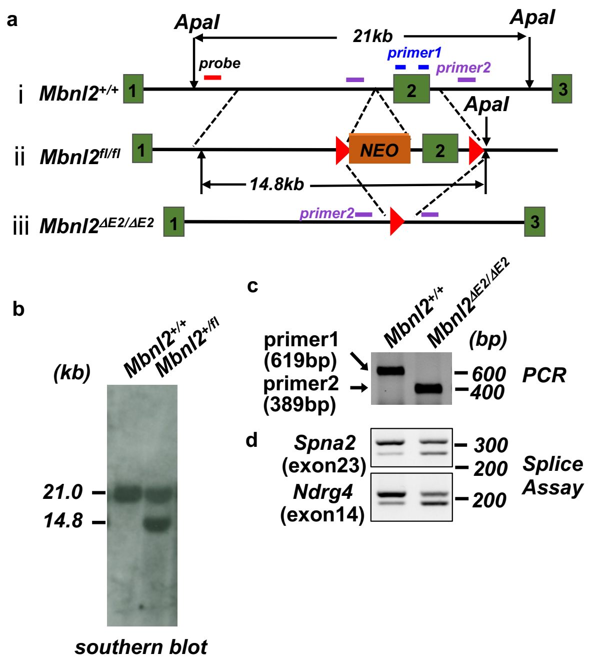

Supplementary Figure 1

Supplementary Figure 1 Legend:

a (i-iii): wild-type *Mbnl2*$^{+/+}$(i), *Mbnl2*$^{fl/fl}$ (ii) and *Mbnl2*$^{ΔE2/ΔE2}$ (iii) alleles are shown. **b:** Southern blot analysis of genomic DNA from targeted 129sv ES cells restricted with ApaI. **c:** PCR analysis of *Mbnl2*$^{+/+}$ and *Mbnl2*$^{ΔE2/ΔE2}$ tail DNA using the indicated primers. **d:** Splice analysis in *Mbnl2*$^{+/+}$ and *Mbnl2*$^{ΔE2/ΔE2}$ forebrains.

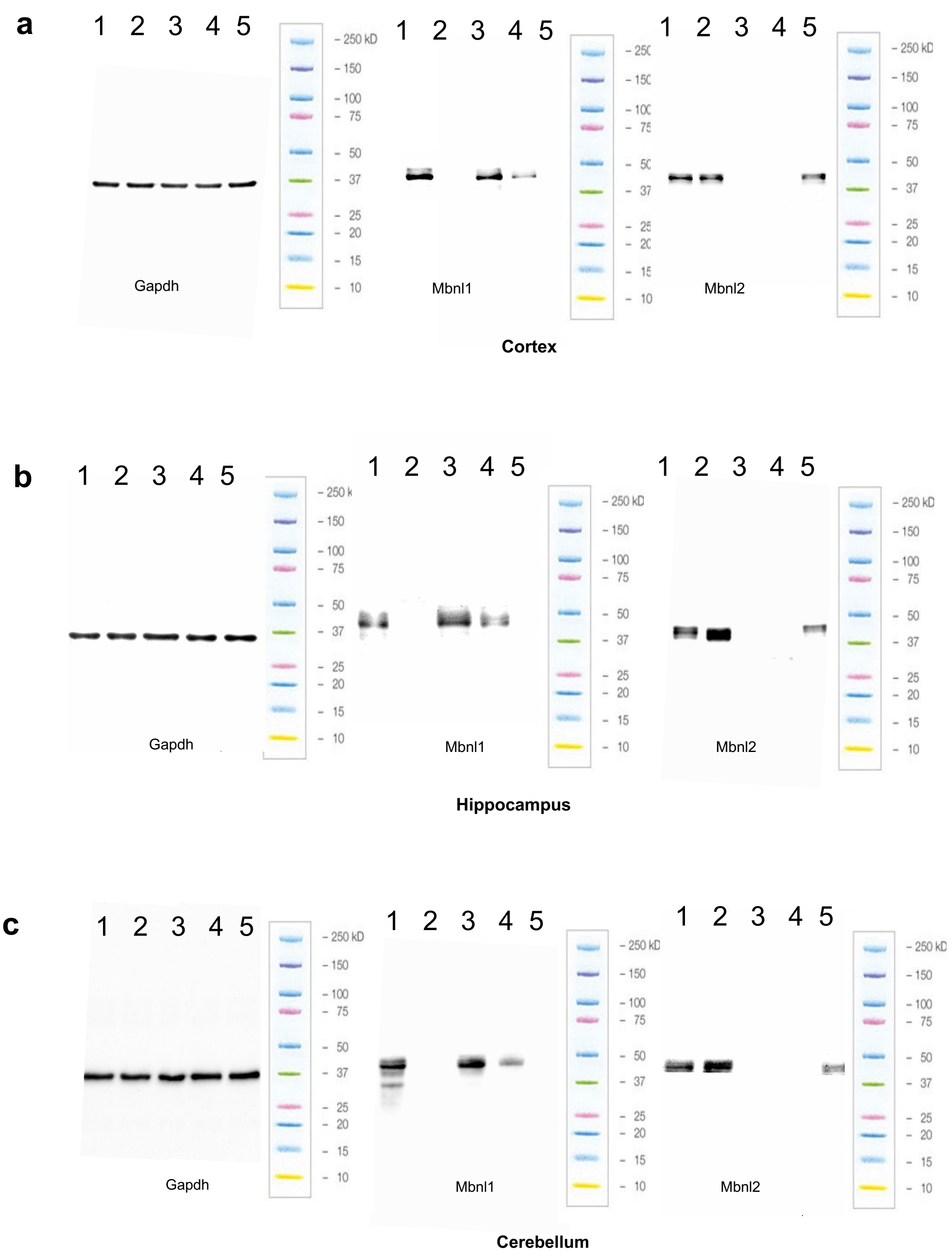
a
1 2 3 4 5
250 kD
150
100
75
50
37
25
20
15
10
Gapdh
Mbnl1
Mbnl2
Cortex
b
Gapdh
Mbnl1
Mbnl2
Hippocampus
c
Gapdh
Mbnl1
Mbnl2
Cerebellum

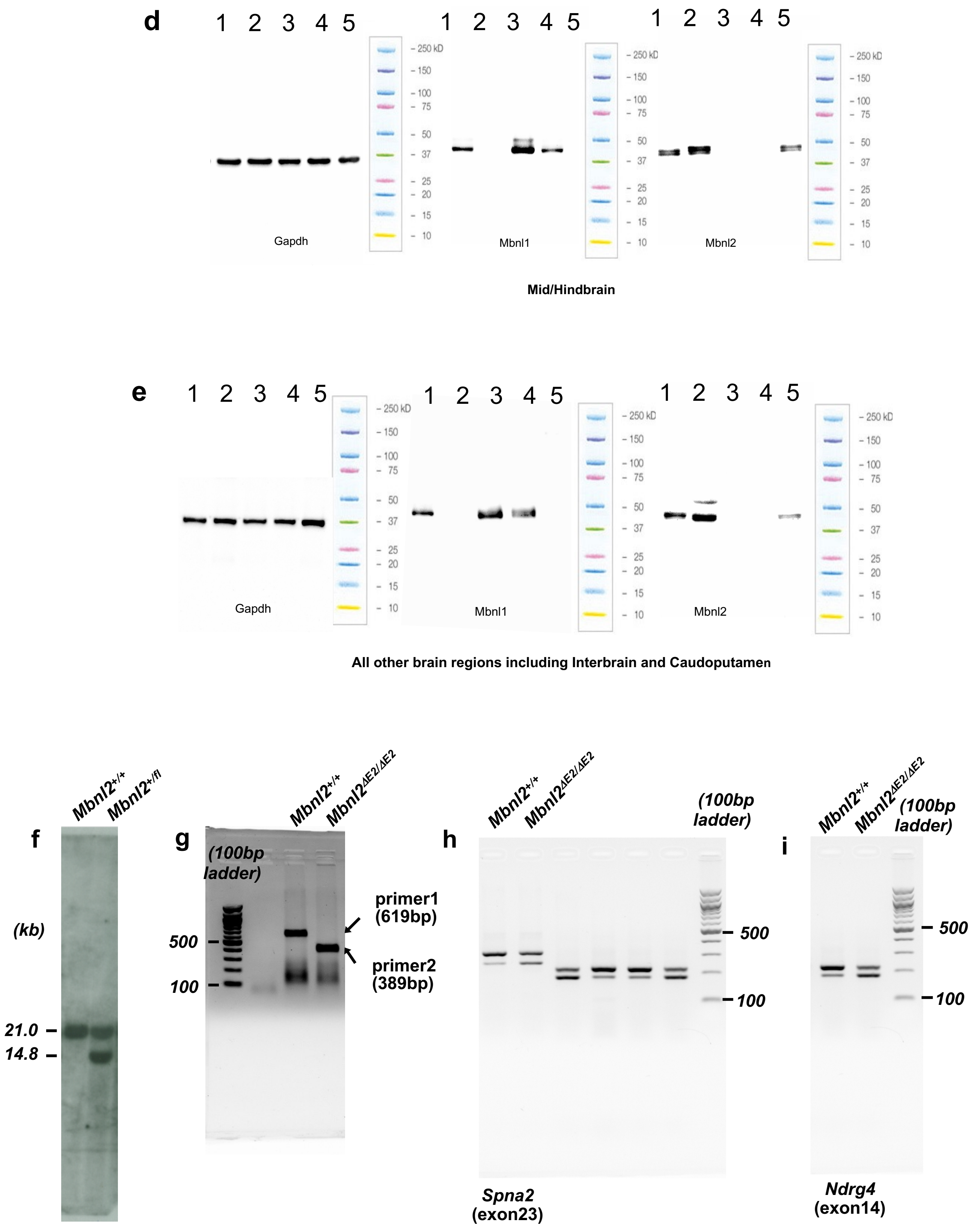

Supplementary Figure 2

Supplementary Figure 2 Legend:

a-e: Uncropped Western blot images shown in Figure 8, b-f. Lane 1: *Mbnl1*$^{+/+}$/*Mbnl2*$^{+/+}$, Lane 2: *Mbnl1*$^{-/-}$, Lane 3: *Mbnl2*$^{-/-}$, Lane 4: *Mbnl1*$^{+/-}$/*Mbnl2*$^{-/-}$, Lane 5: *Mbnl1*$^{-/-}$/*Mbnl2*$^{+/-}$. **f**: Uncropped Southern blot shown in Supplementary Figure 1, b. **g**: Uncropped gel image of PCR analysis of *Mbnl2*$^{+/+}$ and *Mbnl2*$^{\Delta E2/\Delta E2}$ tail clip DNA shown in Supplementary Figure 1, c. **h & i:** Uncropped gel images of splice analysis in *Mbnl2*$^{+/+}$ and *Mbnl2*$^{\Delta E2/\Delta E2}$ forebrains shown in Supplementary Figure 1, d.

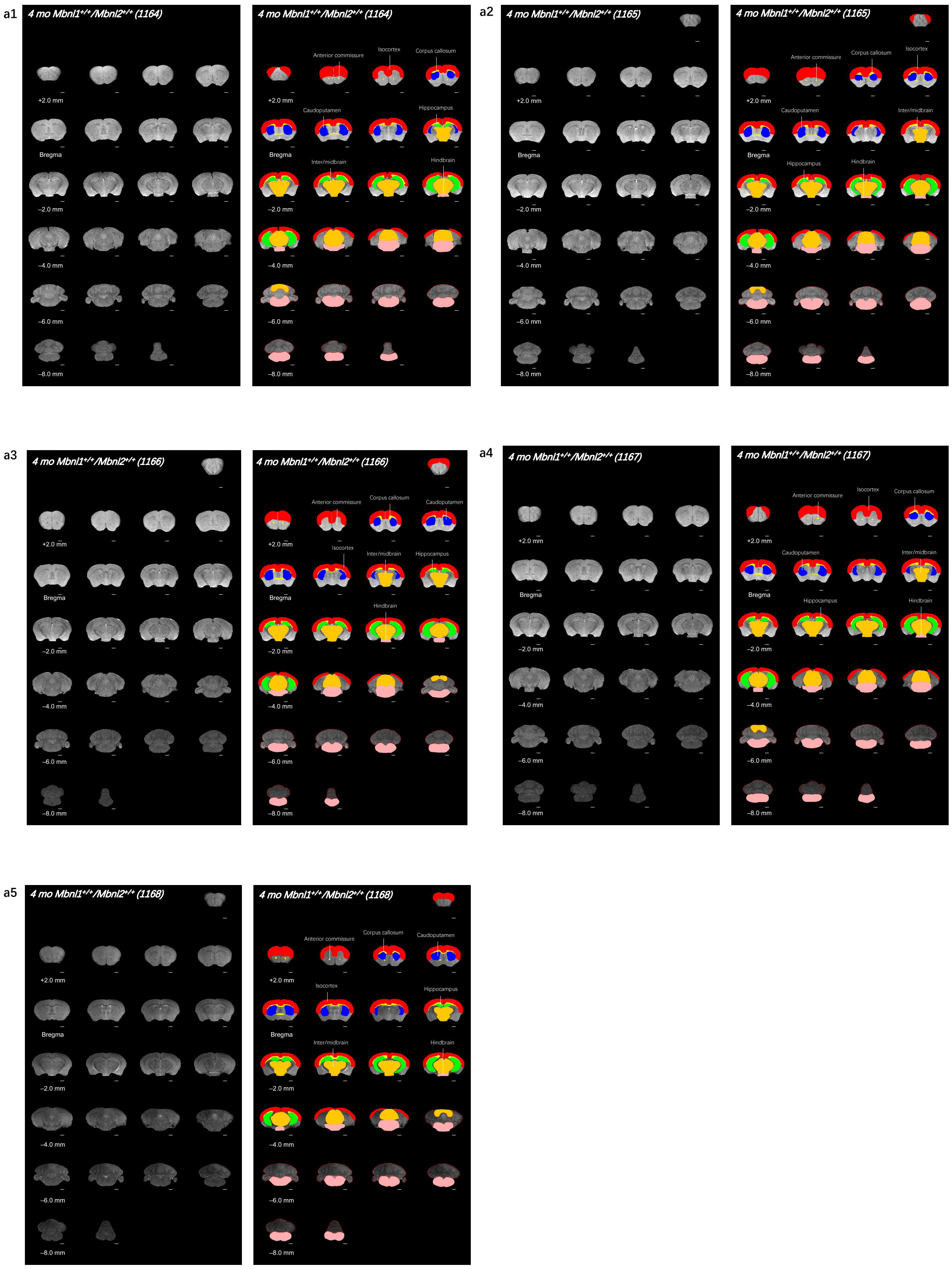

Supplementary Figure 3

Supplementary Figure 3 Legend:

2D FSE T2v *in vivo* MRI scans of 4-month-old *Mbnl1*$^{+/+}$/*Mbnl2*$^{+/+}$ mice.

In all cases individual mouse identifier numbers are shown in parenthesis. In all panels the left montage shows slices of the extracted whole brain that underwent 2D FSE T2w MRI and the right montage shows regions of interests (ROI) manually drawn and color filled over the MRI slices as described in the Methods. Colored regions are white matter regions, which include the anterior commissure and the corpus callosum/external capsule (yellow), and gray matter regions, which include the isocortex or the cerebellum (red), the caudoputamen (blue), the hippocampus (green), the inter/midbrain (orange) and the hindbrain (pink). Only ROIs that are significantly different from the control age matched *Mbnl1*$^{+/+}$/*Mbnl2*$^{+/+}$ brains are color filled in the *Mbnl1*$^{+/+}$/*Mbnl2*$^{+/+}$, *Mbnl1*$^{+/-}$/*Mbnl2*$^{-/-}$, *Mbnl1*$^{-/-}$, *Mbnl2*$^{-/-}$ and *Mbnl1*$^{-/-}$/*Mbnl2*$^{+/-}$ montages. Calculated areas from the ROIs were multiplied by the image slice thickness to generate ROI volumes. The olfactory bulb was excluded in volume measurements. Slice positions were identified based on Bregma locations. The slice where the anterior commissure is connected between hemispheres approximates Bregma=0.0mm. Slice thickness = 0.5 mm. Scale bar = 1 mm.

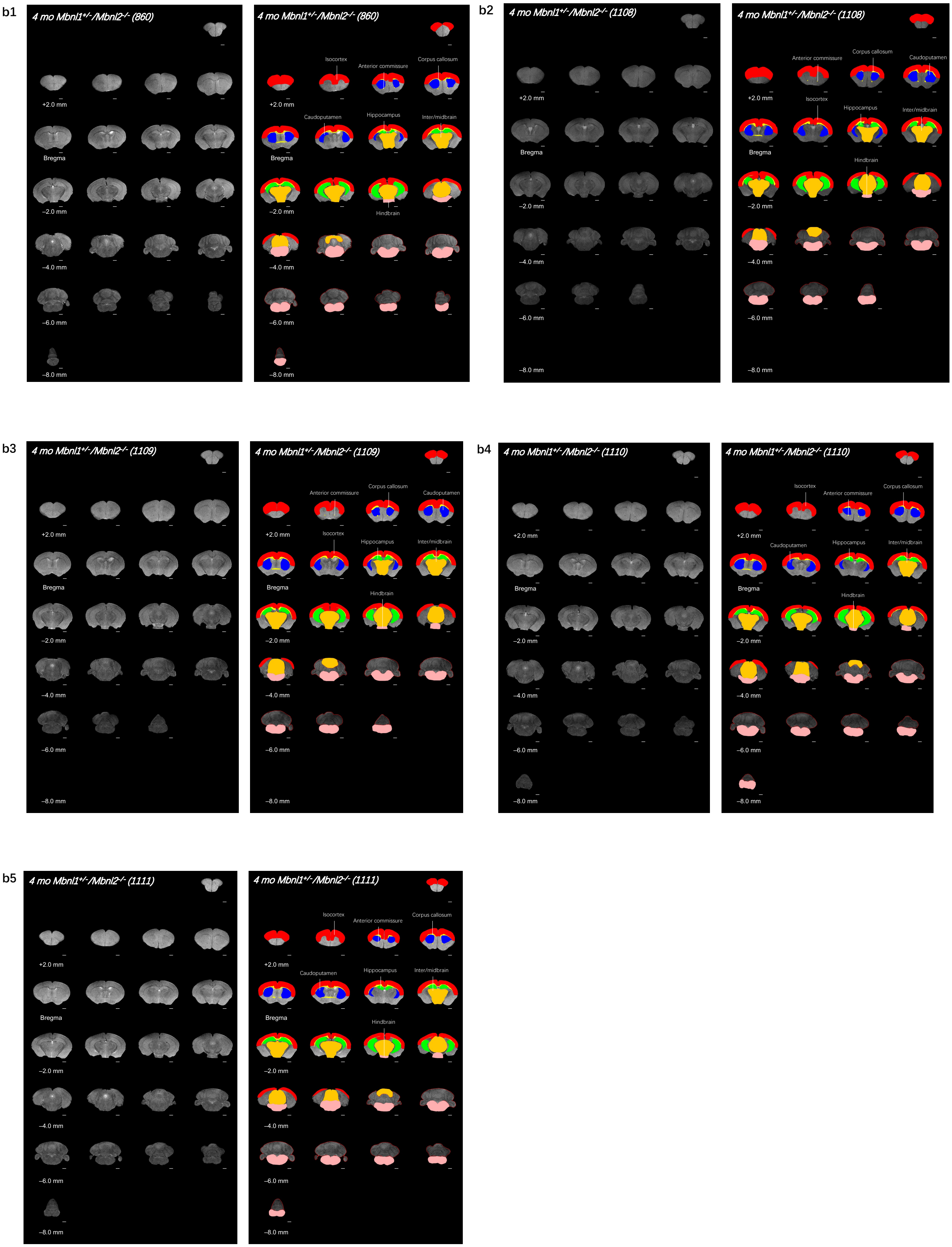

Supplementary Figure 4

Supplementary Figure 4 Legend:

2D FSE T2v *in vivo* MRI scans of 4-month-old *Mbnl1*$^{+/-}$*/Mbnl2*$^{-/-}$ mice. In all cases individual mouse identifier numbers are shown in parenthesis. In all panels the left montage shows slices of the extracted whole brain that underwent 2D FSE T2w MRI and the right montage shows regions of interests (ROI) manually drawn and color filled over the MRI slices as described in the Methods. Colored regions are white matter regions, which include the anterior commissure and the corpus callosum/external capsule (yellow), and gray matter regions, which include the isocortex or the cerebellum (red), the caudoputamen (blue), the hippocampus (green), the inter/midbrain (orange) and the hindbrain (pink). Only ROIs that are significantly different from the control age matched *Mbnl1*$^{+/+}$*/Mbnl2*$^{+/+}$ brains are color filled in the *Mbnl1*$^{+/+}$*/Mbnl2*$^{+/+}$, *Mbnl1*$^{+/-}$*/Mbnl2*$^{-/-}$, *Mbnl1*$^{-/-}$, *Mbnl2*$^{-/-}$ and *Mbnl1*$^{-/-}$*/Mbnl2*$^{+/-}$ montages. Calculated areas from the ROIs were multiplied by the image slice thickness to generate ROI volumes. The olfactory bulb was excluded in volume measurements. Slice positions were identified based on Bregma locations. The slice where the anterior commissure is connected between hemispheres approximates Bregma=0.0mm. Slice thickness = 0.5 mm. Scale bar = 1 mm.

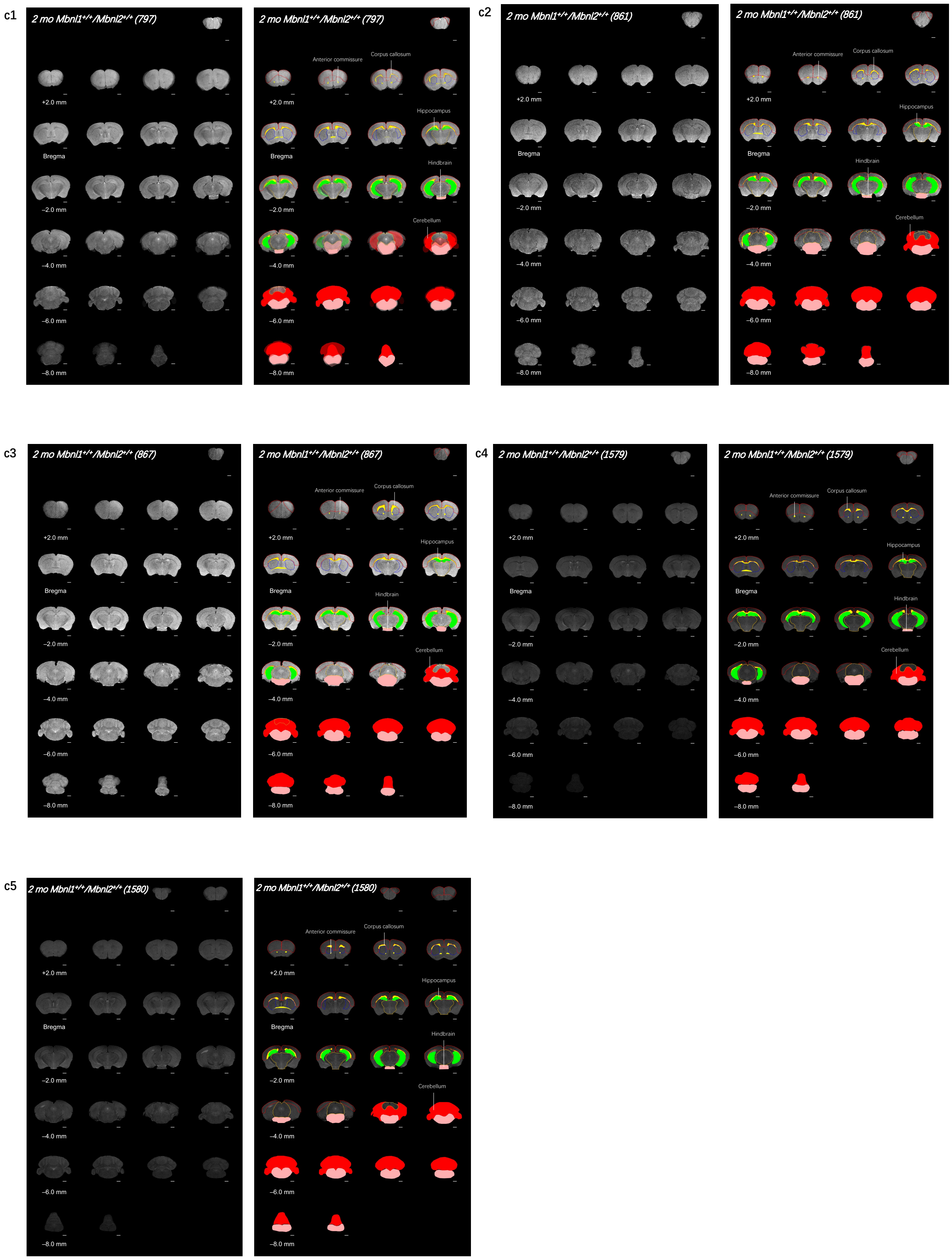

Supplementary Figure 5

Supplementary Figure 5 Legend:

2D FSE T2v *in vivo* MRI scans of 2-month-old *Mbnl1*$^{+/+}$*/Mbnl2*$^{+/+}$ mice. In all cases individual mouse identifier numbers are shown in parenthesis. In all panels the left montage shows slices of the extracted whole brain that underwent 2D FSE T2w MRI and the right montage shows regions of interests (ROI) manually drawn and color filled over the MRI slices as described in the Methods. Colored regions are white matter regions, which include the anterior commissure and the corpus callosum/external capsule (yellow), and gray matter regions, which include the isocortex or the cerebellum (red), the caudoputamen (blue), the hippocampus (green), the inter/midbrain (orange) and the hindbrain (pink). Only ROIs that are significantly different from the control age matched *Mbnl1*$^{+/+}$*/Mbnl2*$^{+/+}$ brains are color filled in the *Mbnl1*$^{+/+}$*/Mbnl2*$^{+/+}$, *Mbnl1*$^{+/-}$*/Mbnl2*$^{-/-}$, *Mbnl1*$^{-/-}$, *Mbnl2*$^{-/-}$ and *Mbnl1*$^{-/-}$*/Mbnl2*$^{+/-}$ montages. Calculated areas from the ROIs were multiplied by the image slice thickness to generate ROI volumes. The olfactory bulb was excluded in volume measurements. Slice positions were identified based on Bregma locations. The slice where the anterior commissure is connected between hemispheres approximates Bregma=0.0mm. Slice thickness = 0.5 mm. Scale bar = 1 mm.

d1

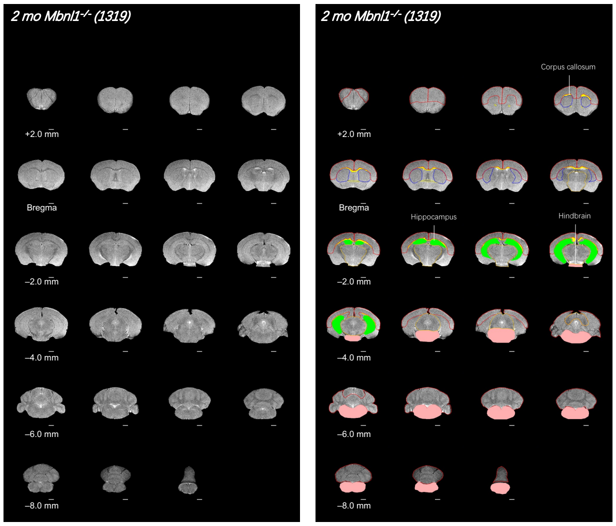

d2

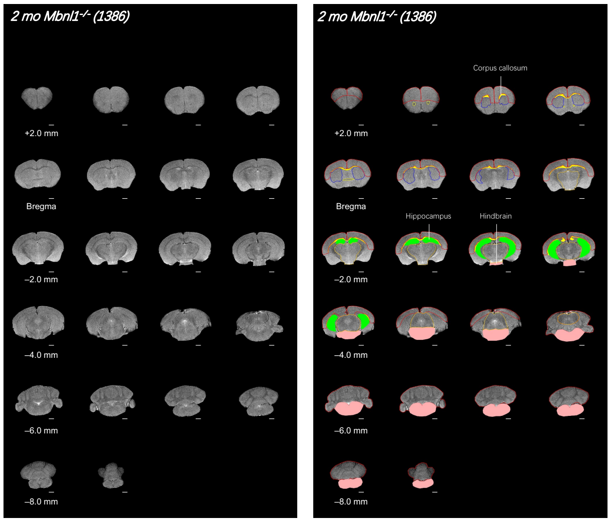

d3

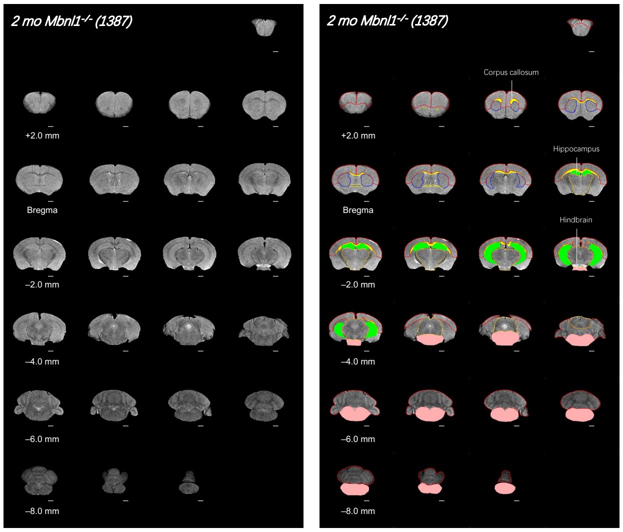

d4

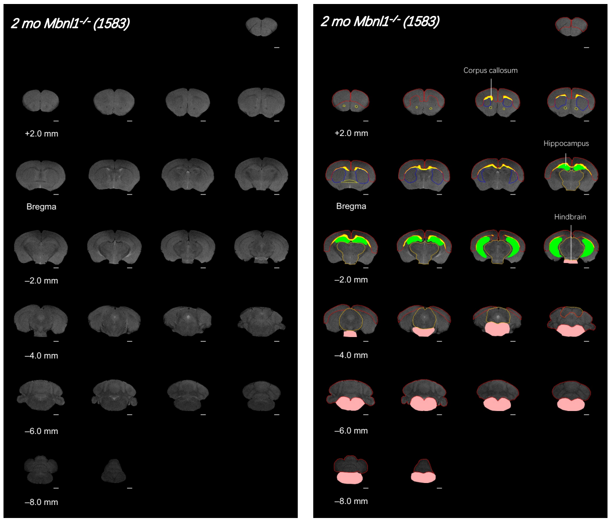

d5

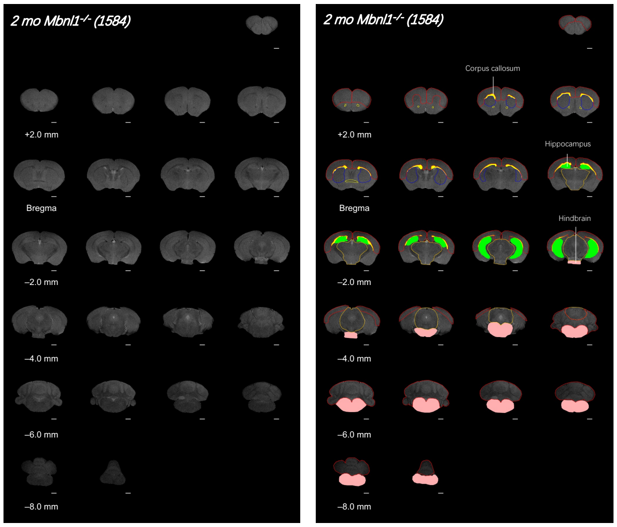

Supplementary Figure 6

Supplementary Figure 6 Legend:

2D FSE T2v *in vivo* MRI scans of 2-month-old *Mbnl1*$^{-/-}$ mice. In all cases individual mouse identifier numbers are shown in parenthesis. In all panels the left montage shows slices of the extracted whole brain that underwent 2D FSE T2w MRI and the right montage shows regions of interests (ROI) manually drawn and color filled over the MRI slices as described in the Methods. Colored regions are white matter regions, which include the anterior commissure and the corpus callosum/external capsule (yellow), and gray matter regions, which include the isocortex or the cerebellum (red), the caudoputamen (blue), the hippocampus (green), the inter/midbrain (orange) and the hindbrain (pink). Only ROIs that are significantly different from the control age matched *Mbnl1*$^{+/+}$/*Mbnl2*$^{+/+}$ brains are color filled in the *Mbnl1*$^{+/+}$/*Mbnl2*$^{+/+}$, *Mbnl1*$^{+/-}$/*Mbnl2*$^{-/-}$, *Mbnl1*$^{-/-}$, *Mbnl2*$^{-/-}$ and *Mbnl1*$^{-/-}$/*Mbnl2*$^{+/-}$ montages. Calculated areas from the ROIs were multiplied by the image slice thickness to generate ROI volumes. The olfactory bulb was excluded in volume measurements. Slice positions were identified based on Bregma locations. The slice where the anterior commissure is connected between hemispheres approximates Bregma=0.0mm. Slice thickness = 0.5 mm. Scale bar = 1 mm.

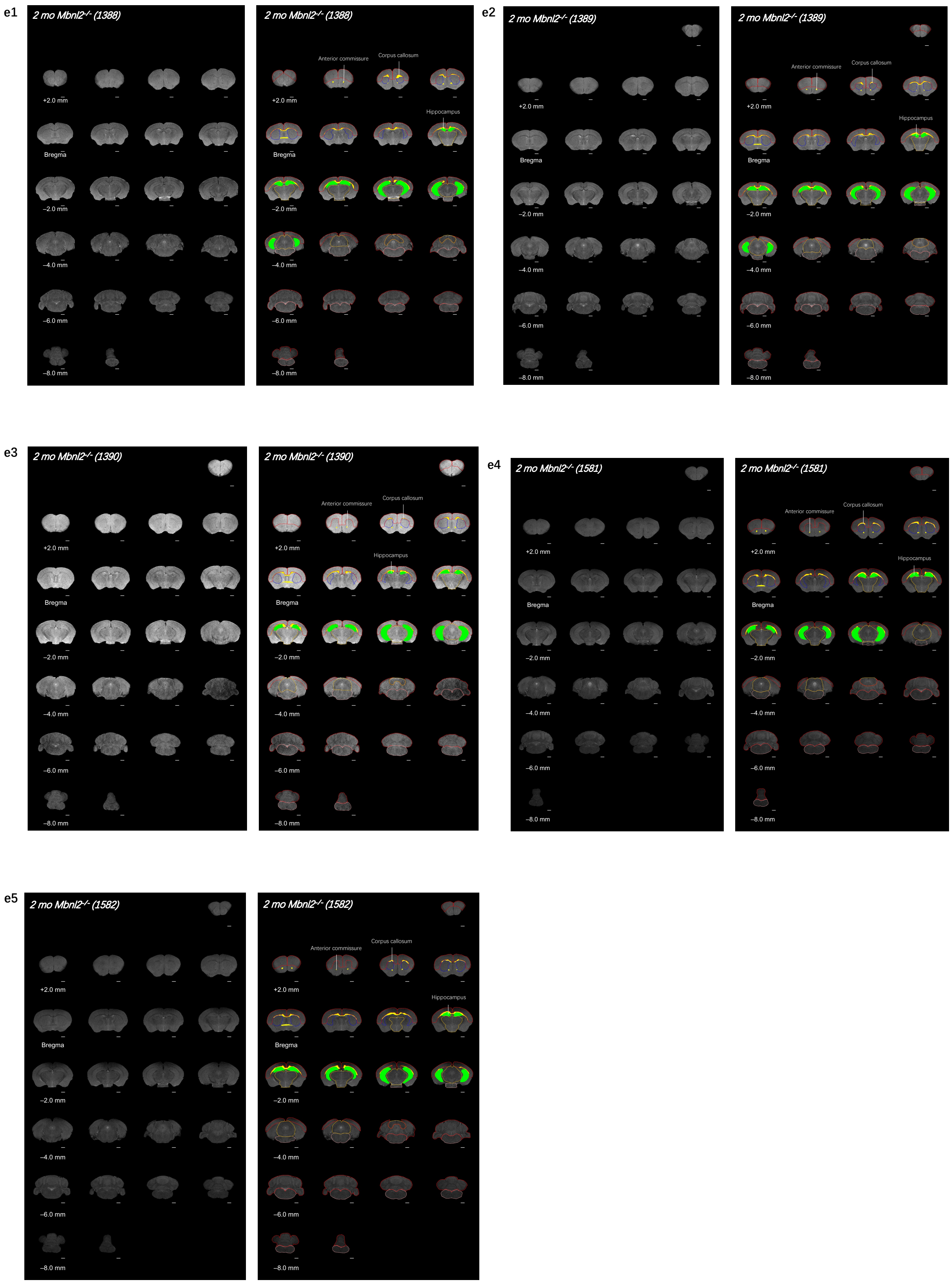

Supplementary Figure 7

Supplementary Figure 7 Legend:

2D FSE T2v *in vivo* MRI scans 2-month-old *Mbnl2*$^{-/-}$ mice. In all cases individual mouse identifier numbers are shown in parenthesis. In all panels the left montage shows slices of the extracted whole brain that underwent 2D FSE T2w MRI and the right montage shows regions of interests (ROI) manually drawn and color filled over the MRI slices as described in the Methods. Colored regions are white matter regions, which include the anterior commissure and the corpus callosum/external capsule (yellow), and gray matter regions, which include the isocortex or the cerebellum (red), the caudoputamen (blue), the hippocampus (green), the inter/midbrain (orange) and the hindbrain (pink). Only ROIs that are significantly different from the control age matched *Mbnl1*$^{+/+}$/*Mbnl2*$^{+/+}$ brains are color filled in the *Mbnl1*$^{+/+}$/*Mbnl2*$^{+/+}$, *Mbnl1*$^{+/-}$/*Mbnl2*$^{-/-}$, *Mbnl1*$^{-/-}$, *Mbnl2*$^{-/-}$ and *Mbnl1*$^{-/-}$/*Mbnl2*$^{+/-}$ montages. Calculated areas from the ROIs were multiplied by the image slice thickness to generate ROI volumes. The olfactory bulb was excluded in volume measurements. Slice positions were identified based on Bregma locations. The slice where the anterior commissure is connected between hemispheres approximates Bregma=0.0mm. Slice thickness = 0.5 mm. Scale bar = 1 mm.

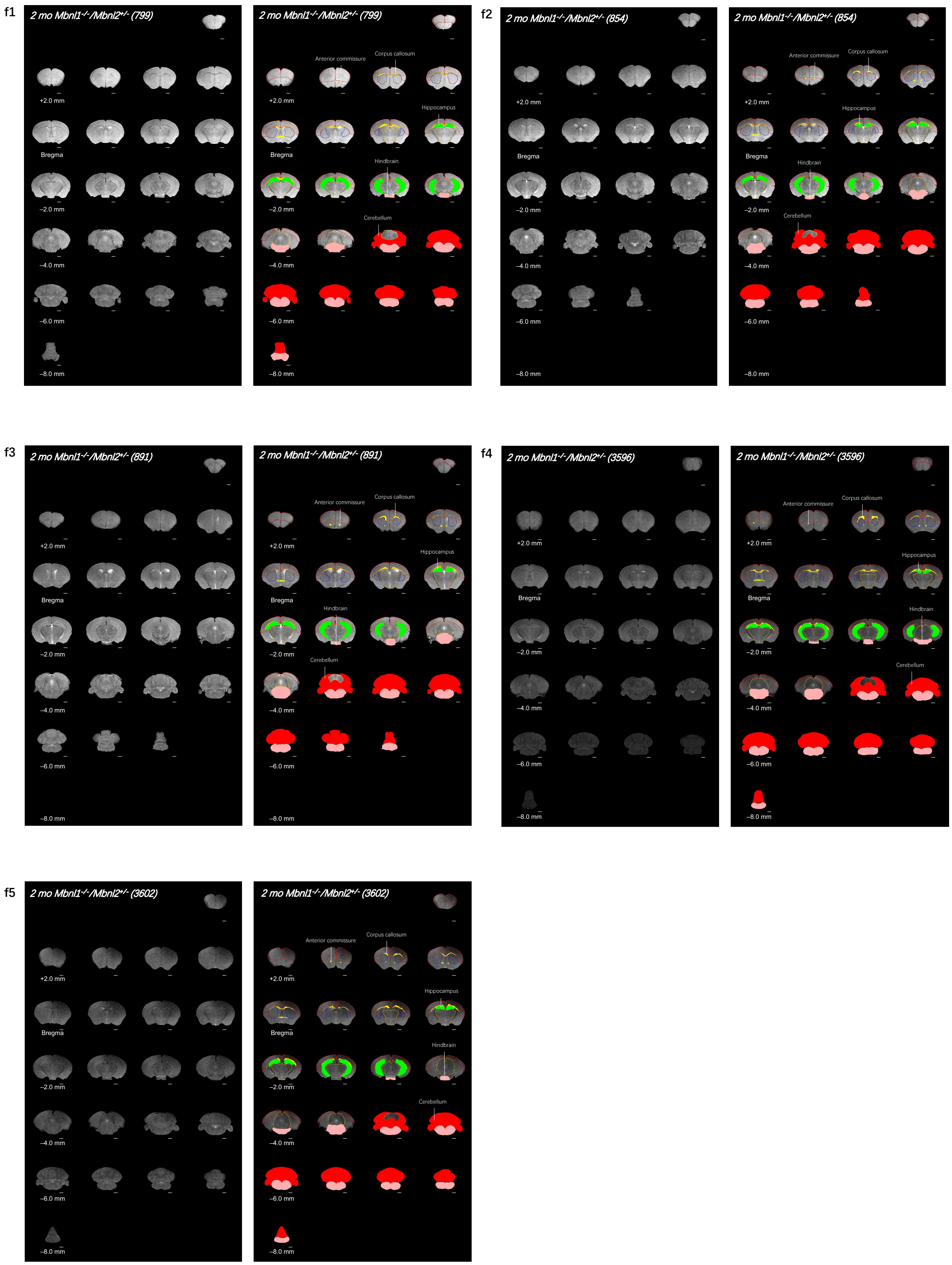

Supplementary Figure 8

Supplementary Figure 8 Legend:

2D FSE T2v *in vivo* MRI scans 2-month-old *Mbnl1*$^{-/-}$*/Mbnl2*$^{+/-}$ mice. In all cases individual mouse identifier numbers are shown in parenthesis. In all panels the left montage shows slices of the extracted whole brain that underwent 2D FSE T2w MRI and the right montage shows regions of interests (ROI) manually drawn and color filled over the MRI slices as described in the Methods. Colored regions are white matter regions, which include the anterior commissure and the corpus callosum/external capsule (yellow), and gray matter regions, which include the isocortex or the cerebellum (red), the caudoputamen (blue), the hippocampus (green), the inter/midbrain (orange) and the hindbrain (pink). Only ROIs that are significantly different from the control age matched *Mbnl1*$^{+/+}$*/Mbnl2*$^{+/+}$ brains are color filled in the *Mbnl1*$^{+/+}$*/Mbnl2*$^{+/+}$, *Mbnl1*$^{+/-}$*/Mbnl2*$^{-/-}$, *Mbnl1*$^{-/-}$, *Mbnl2*$^{-/-}$ and *Mbnl1*$^{-/-}$*/Mbnl2*$^{+/-}$ montages. Calculated areas from the ROIs were multiplied by the image slice thickness to generate ROI volumes. The olfactory bulb was excluded in volume measurements. Slice positions were identified based on Bregma locations. The slice where the anterior commissure is connected between hemispheres approximates Bregma=0.0mm. Slice thickness = 0.5 mm. Scale bar = 1 mm.

Supplementary Reference:

1. O'Gorman, S., Dagenais, N.A., Qian, M. & Marchuk, Y. Protamine-Cre recombinase transgenes efficiently recombine target sequences in the male germ line of mice, but not in embryonic stem cells. *Proc Natl Acad Sci U S A* **94**,14602-14607 (1997).
