# Supplementary material for: Mbnl1 and Mbnl2 regulate brain structural integrity in mice
Source: Commun Biol. 2021 Nov 30;4:1342. doi: 10.1038/s42003-021-02845-0 (PMC8633067; doi:10.1038/s42003-021-02845-0)
Supplement: Supplementary file 3 — Description of Additional Supplementary Files [file 42003_2021_2845_MOESM3_ESM.pdf]

Description of Additional Supplementary Files

File name: Supplementary Data 1.
Description: MRI source data.
