# Supplementary material for: Mbnl1 and Mbnl2 regulate brain structural integrity in mice
Source: Commun Biol. 2021 Nov 30;4:1342. doi: 10.1038/s42003-021-02845-0 (PMC8633067; doi:10.1038/s42003-021-02845-0)
Supplement: Supplementary file 5 — Reporting Summary [file 42003_2021_2845_MOESM5_ESM.pdf]

nature research

Corresponding author(s): Reddy, Comai

Last updated by author(s): 2021-9-16

Reporting Summary

Nature Research wishes to improve the reproducibility of the work that we publish. This form provides structure for consistency and transparency in reporting. For further information on Nature Research policies, see our Editorial Policies and the Editorial Policy Checklist.

Statistics

For all statistical analyses, confirm that the following items are present in the figure legend, table legend, main text, or Methods section.

n/a	Confirmed	
☐	☒	The exact sample size (n) for each experimental group/condition, given as a discrete number and unit of measurement
☐	☒	A statement on whether measurements were taken from distinct samples or whether the same sample was measured repeatedly
☐	☒	The statistical test(s) used AND whether they are one- or two-sided<br>*Only common tests should be described solely by name; describe more complex techniques in the Methods section.*
☒	☐	A description of all covariates tested
☐	☒	A description of any assumptions or corrections, such as tests of normality and adjustment for multiple comparisons
☐	☒	A full description of the statistical parameters including central tendency (e.g. means) or other basic estimates (e.g. regression coefficient) AND variation (e.g. standard deviation) or associated estimates of uncertainty (e.g. confidence intervals)
☐	☒	For null hypothesis testing, the test statistic (e.g. F, t, r) with confidence intervals, effect sizes, degrees of freedom and P value noted<br>*Give P values as exact values whenever suitable.*
☒	☐	For Bayesian analysis, information on the choice of priors and Markov chain Monte Carlo settings
☒	☐	For hierarchical and complex designs, identification of the appropriate level for tests and full reporting of outcomes
☒	☐	Estimates of effect sizes (e.g. Cohen's d, Pearson's r), indicating how they were calculated

Our web collection on statistics for biologists contains articles on many of the points above.

Software and code

Policy information about availability of computer code

Data collection	MRSolutions, Inc PreClinicalScan 4.0.2.31
Data analysis	GraphPad Prism 9 (San Diego, CA, USA) statistical software was used.

For manuscripts utilizing custom algorithms or software that are central to the research but not yet described in published literature, software must be made available to editors and reviewers. We strongly encourage code deposition in a community repository (e.g. GitHub). See the Nature Research guidelines for submitting code & software for further information.

Data

Policy information about availability of data

All manuscripts must include a data availability statement. This statement should provide the following information, where applicable:

- Accession codes, unique identifiers, or web links for publicly available datasets
- A list of figures that have associated raw data
- A description of any restrictions on data availability

All source data are shown in Supplementary Figures 2 &3 and Supplementary Table 1. No custom codes or mathematical algorithms were used in these analyses. All procedures are approved by the Institutional Animal Care and Use Committee at the University of Southern California. Transgenic animals are available for non-commercial purposes from S.R.

Field-specific reporting

Please select the one below that is the best fit for your research. If you are not sure, read the appropriate sections before making your selection.

☒ Life sciences ☐ Behavioural & social sciences ☐ Ecological, evolutionary & environmental sciences

For a reference copy of the document with all sections, see nature.com/documents/nr-reporting-summary-flat.pdf

Life sciences study design

All studies must disclose on these points even when the disclosure is negative.

Sample size	All mouse groups (both 4 and 2-months) have 5 mice/genotype. We have calculated the percent change we would be able to detect with 5 animals per group (n=10) based on a one-sample test for proportions. Assuming that, under the null hypothesis, the percent change is 1%, we would be able to detect a change of 14.4% or higher with 80% power at a Type I error rate of 5%. Similarly, for the 2-month old mice, we will be making pairwise comparisons between selected groups and would therefore be able to detect a similar percent change between any two groups. The relatively small standard deviations between subjects in some cases however allowed us to pick up significant differences ranging from ~6 to ~50% in these cohorts, which are similar to effect sizes observed in DM1 patients (3,4,22-28 & Kassubek, J et al. Quantification of brain atrophy in patients with myotonic dystrophy and proximal myotonic myopathy: a controlled 3-dimensional magnetic resonance imaging study. Neuroscience Letters 348, 73–76 (2003), Antonini, G. et al. Cerebral atrophy in myotonic dystrophy: a voxel based morphometric study. J Neurol Neurosurg Psychiatry 75, 1611–1613 (2004)].
Data exclusions	None
Replication	All studies were conducted in a single cohort of animals as described. Replication was not attempted.
Randomization	For all studies genotyping determined allocation to the experimental group.
Blinding	Three blinded experimenters (NS, CZ, and SJ) independently drew all ROIs and calculated volumes. Statistical analysis of volume measurements demonstrated no significant differences between the experimenters. Discussions were held by the experimenters to reach an unequivocal consensus on disparate measurements.

Reporting for specific materials, systems and methods

We require information from authors about some types of materials, experimental systems and methods used in many studies. Here, indicate whether each material, system or method listed is relevant to your study. If you are not sure if a list item applies to your research, read the appropriate section before selecting a response.

Materials & experimental systems

n/a	Involved in the study	
☐	☒	Antibodies
☒	☐	Eukaryotic cell lines
☒	☐	Palaeontology and archaeology
☐	☒	Animals and other organisms
☒	☐	Human research participants
☒	☐	Clinical data
☒	☐	Dual use research of concern

Methods

n/a	Involved in the study	
☒	☐	ChIP-seq
☒	☐	Flow cytometry
☐	☒	MRI-based neuroimaging

Antibodies

Antibodies used	Anti-Gapdh and anti-Mbnl2 antibodies were purchased from Santa Cruz Biotechnology (Gapdh, Cat # sc-32233, Lot #K3016, 100ug/ml, dilution 1:200; Mbnl2 Cat # sc-136167, Lot # D0417, 200ug/ml, dilution 1:200 (monoclonal antibody raised against recombinant human Mbnl2), anti-Mbnl1 antibodies are a gift from Ian Holt, dilution 1:100 Ref 42.
Validation	Anti-Mbnl1 and anti-Mbnl2 antibodies were validated in Mbnl1-/- and Mbnl2-/- brains.

Animals and other organisms

Policy information about studies involving animals; ARRIVE guidelines recommended for reporting animal research

Laboratory animals	The following mouse strains were used in this study: 129sv Mbnl1ΔE2/ΔE2 mice (indicated as Mbnl1-/-, where the ATG encoding Mbnl1 exon 2 is deleted) (Ref 32). 129sv Mbnl2ΔE2/ΔE2 mice (indicated as Mbnl2-/-, where the ATG encoding Mbnl2 exon 2 is deleted) (Supplementary Figure 1). These strains were bred to develop 129sv Mbnl1ΔE2/ΔE2/ Mbnl2+/ΔE2 and 129sv Mbnl1+/ΔE2/ Mbnl2 ΔE2/ΔE2 mice denoted here as Mbnl1-/-/Mbnl2+/- and Mbnl1+/-/Mbnl2-/- mice, respectively. Age and gender matched

	wildtype 129sv mice (indicated as Mbnl1+/+/Mbnl2+/+) were used as controls. For all groups n = 5 mice.
Wild animals	*Provide details on animals observed in or captured in the field; report species, sex and age where possible. Describe how animals were caught and transported and what happened to captive animals after the study (if killed, explain why and describe method; if released, say where and when) OR state that the study did not involve wild animals.*
Field-collected samples	*For laboratory work with field-collected samples, describe all relevant parameters such as housing, maintenance, temperature, photoperiod and end-of-experiment protocol OR state that the study did not involve samples collected from the field.*
Ethics oversight	All procedures are approved by the Institutional Animal Care and Use Committee at the University of Southern California.

Note that full information on the approval of the study protocol must also be provided in the manuscript.

Magnetic resonance imaging

Experimental design

Design type	*Indicate task or resting state; event-related or block design.*
Design specifications	*Specify the number of blocks, trials or experimental units per session and/or subject, and specify the length of each trial or block (if trials are blocked) and interval between trials.*
Behavioral performance measures	*State number and/or type of variables recorded (e.g. correct button press, response time) and what statistics were used to establish that the subjects were performing the task as expected (e.g. mean, range, and/or standard deviation across subjects).*

Acquisition

Imaging type(s)	anatomical
Field strength	7T
Sequence & imaging parameters	Two-dimensional (2D) Fast Spin Echo (FSE) T2-weighted sequence was used to define neuroanatomy in the transverse axial orientation. FSE T2w scan parameters were as follows: TE=45 ms, TR = 4000 ms, number of averages = 4, echo train length = 7, field of view = 14 mm x 14 mm, slice thickness = 0.5 mm, number of slices = 28, and matrix size = 256 x 256.
Area of acquisition	The whole brain image was acquired excluding the olfactory bulb.

Diffusion MRI ☐ Used ☒ Not used

Preprocessing

Preprocessing software	No preprocessing software was used.
Normalization	*If data were normalized/standardized, describe the approach(es): specify linear or non-linear and define image types used for transformation OR indicate that data were not normalized and explain rationale for lack of normalization.*
Normalization template	*Describe the template used for normalization/transformation, specifying subject space or group standardized space (e.g. original Talairach, MNI305, ICBM152) OR indicate that the data were not normalized.*
Noise and artifact removal	*Describe your procedure(s) for artifact and structured noise removal, specifying motion parameters, tissue signals and physiological signals (heart rate, respiration).*
Volume censoring	*Define your software and/or method and criteria for volume censoring, and state the extent of such censoring.*

Statistical modeling & inference

Model type and settings	Two tailed t-test was performed when comparing the ROI volumes (mean + SD) of 4-month-old Mbnl1+/+/Mbnl2+/+ and Mbnl1+/-/Mbnl2-/- cohorts. One-way ANOVA analysis was performed when comparing the ROI volumes (mean + SD) of 2-month-old Mbnl1+/+/Mbnl2+/+, Mbnl1-/-, Mbnl2-/- and Mbnl1-/-/Mbnl2+/- mice, followed by the Dunnett's multiple comparisons test. In some cases, the two tailed t-test was performed when comparing genotypes in the 2-month-old cohorts.
Effect(s) tested	*Define precise effect in terms of the task or stimulus conditions instead of psychological concepts and indicate whether ANOVA or factorial designs were used.*

Specify type of analysis: ☐ Whole brain ☐ ROI-based ☒ Both

Anatomical location(s)	Regions of interests (ROI) were manually defined on the 2D T2w FSE MRI scans using the polygon tool in ImageJ software (40) and using Hof et al. (41) and the Allen mouse brain atlas as visual guides (http://mouse.brain-map.org/). ROI area measurements for each slice were multiplied by the image slice thickness (0.5 mm) and summed to generate volumes. Three blinded experimenters (NS, CZ, and SJ) independently drew all ROIs and calculated volumes. Statistical analysis of volume measurements demonstrated no significant differences between the experimenters. Discussions were held by the experimenters to reach an unequivocal consensus on disparate measurements.<br><br>The following white and gray matter ROIs were measured: defined white matter regions included the anterior commissure and the corpus callosum/external capsule. Gray matter regions included the cerebrum (isocortex, hippocampus proper and subiculum, denoted as hippocampus and caudoputamen), brain stem (inter/mid brain and hind brain) and the cerebellum. Whole brain and apparent ventricle volumes were measured. Apparent ventricle volumes were delineated as visibly identifiable ventricle regions with hyperintense signals in the FSE T2w brain slices. The olfactory bulb was excluded in volume measurements.
Statistic type for inference<br>(See Eklund et al. 2016)	*Specify voxel-wise or cluster-wise and report all relevant parameters for cluster-wise methods.*
Correction	*Describe the type of correction and how it is obtained for multiple comparisons (e.g. FWE, FDR, permutation or Monte Carlo).*

Models & analysis

n/a	Involved in the study
☒	☐ Functional and/or effective connectivity
☒	☐ Graph analysis
☒	☐ Multivariate modeling or predictive analysis
